# Maternal Serotonergic Antidepressant Use in Pregnancy and Risk of Seizures in Children

**DOI:** 10.1212/WNL.0000000000200516

**Published:** 2022-06-07

**Authors:** Kelsey Kathleen Wiggs, Ayehsa C. Sujan, Martin E. Rickert, Patrick D. Quinn, Henrik Larsson, Paul Lichtenstein, Brian M. D'Onofrio, A. Sara Oberg

**Affiliations:** From the Department of Psychological & Brain Sciences (K.K.W., M.E.R., B.M.D.) and Department of Applied Health Science, School of Public Health (P.D.Q.), Indiana University, Bloomington; Kaiser Permanente Northern California Division of Research (A.C.S.), Oakland; Department of Medical Epidemiology and Biostatistics (H.L., P.L., B.M.D., A.S.O.), Karolinska Institutet, Stockholm; School of Medical Sciences (H.L.), Örebro University, Sweden; and Department of Epidemiology (A.S.O.), T.H. Chan School of Public Health, Harvard, Boston, MA.

## Abstract

**Background and Objectives:**

To evaluate whether children born to women who use serotonergic antidepressants during pregnancy have higher risk of neonatal seizures and epilepsy.

**Methods:**

We used Swedish register-based data to examine associations between maternal reported use of selective serotonin reuptake inhibitors (SSRIs) or serotonin–norepinephrine reuptake inhibitors (SNRIs) in pregnancy and diagnosis of neonatal seizures or epilepsy in >1.2 million children. To account for systematic differences between exposed and unexposed children, we adjusted for a wide range of measured confounders. After first evaluating the role of maternal indication for SSRI/SNRI use (i.e., depression or anxiety) and parental epilepsy, we adjusted for remaining parental background factors (e.g., age, comorbidities, education, and family socioeconomic indices) and pregnancy-specific characteristics (e.g., maternal use of other psychotropic medications and tobacco smoking in early pregnancy).

**Results:**

Compared with all other children, children of women who reported use of SSRI/SNRI in pregnancy had an elevated risk of neonatal seizures and epilepsy (risk ratio [RR] 1.41, 95% CI 1.03–1.94; hazard ratio [HR] 1.21, 95% CI 1.03–1.43, respectively). The estimates of association were attenuated by adjustment for maternal indications for SSRI/SNRI use (RR 1.30, 95% CI 0.94–1.80; HR 1.13, 95% CI 0.95–1.33), but not by additional adjustment for parental history of epilepsy. Full adjustment for all measured parental and pregnancy-specific factors resulted in substantial attenuation of the remaining associations (RR 1.10, 95% CI 0.79–1.53; HR 0.96, 95% CI 0.81–1.14).

**Discussion:**

We found no support for the concern that maternal SSRI/SNRI use in pregnancy increases children's risk for neonatal seizures or epilepsy.

**Classification of Evidence:**

This study provides Class II evidence that exposure to SSRIs/SNRIs in the first trimester of pregnancy is not associated with an increased incidence of neonatal seizures/epilepsy.

Several studies have documented an association between serotonergic antidepressant (i.e., selective serotonin reuptake inhibitor [SSRI] or serotonin–norepinephrine reuptake inhibitor [SNRI]) use in pregnancy and neonatal seizures, particularly third trimester use.^1-13^ Few studies, however, have examined whether these medications are associated with recurrent seizures (i.e., epilepsy) in childhood.^14^

Given that seizures in neonates can be observed as part of neonatal abstinence syndrome^1-13^ just after antidepressant supply is cut off, it is possible that observed risks are the result of withdrawal. Prenatal exposure to serotonergic antidepressants may also disrupt synaptogenesis and neuronal growth and differentiation^14-17^ in such a way that could influence the risk of longer-term, recurrent seizures.

However, before concluding that observed associations are causal, limitations to the extant literature must be addressed. Given the rarity of seizures, most previous studies have been underpowered.^1,3,4,6,8,10-13,18^ In addition, as noted in a recent review, the existing research provides correlational evidence without adjustment for important confounders, including the maternal indication for antidepressant use, which could be comorbid with seizure disorders (e.g., maternal depression and anxiety).^19-22^ Thus, it is unclear whether observed risks of seizures in children could be due to prenatal pharmacologic exposure or confounding by maternal background factors.

We aimed to evaluate whether children born to women who use serotonergic antidepressants during pregnancy have higher risk of neonatal seizures and epilepsy in a nationwide sample of children. We considered many confounders not incorporated in prior studies (i.e., parental characteristics, sociodemographics, pregnancy-specific factors). We adjusted for psychiatric and behavioral health problems because there has been well-documented comorbidity between these conditions (particularly depression) and seizures that is likely to be at least partially genetic in origin.^20-22,29,30^ As such, it stands to reason that parents with psychiatric and behavioral problems may be more likely to have children with seizures. We further sought to expand the previous literature by also examining the potential connection to children's risk of developing epilepsy.

## Methods

### Standard Protocol Approvals, Registrations, and Patient Consents

The institutional review board at Indiana University and the regional ethical review board in Stockholm, Sweden, approved this study. According to Swedish law, informed consent was not necessary because the study used data available from national Swedish registries.

### Data Availability

The data used in this study are national register information. The authors had no privileges in accessing the data. Dissemination of personal information is regulated by the Swedish Secrecy Act. In accordance with Swedish law, researchers seeking access to individual-level data must apply for permission through a Research Ethics Board and from the primary owners: Statistics Sweden and the National Board of Health and Welfare.

### Data Source

Each individual in Sweden is assigned a unique registration number through which records of health and demographics in national registers can be linked to follow individuals over time. The current study centered on information from the Medical Birth Register, which includes data from antenatal visits, delivery, and pediatric examination for approximately 98% of all births since 1973. Since mid-1994, women have reported any use of medications as part of the standardized interview at enrollment to antenatal care, which typically occurs between weeks 8 and 12 of pregnancy.^23,24^ At this time the midwife also collects information regarding the woman's age, cohabitation status, reproductive history, and use of tobacco. From mid-2005, the maternal self-report of medication use can be evaluated against the filling of prescriptions using the Swedish Prescribed Drug Register (PDR) of all filled prescriptions outside hospital.^25^ Fathers were identified using the Multi-Generation Register.^26^ The National Patient Register includes ICD-coded diagnoses made during all inpatient visits since 1987 and at outpatient specialist visits since 2001.^27^ We used this register to identify parental epilepsy and psychiatric diagnoses (including depression and anxiety) as well as neonatal seizure and epilepsy diagnoses in children. Finally, we used the Integrated Database for Labor Market Research^28^ and the Education Register to retrieve information on socioeconomic indices.

### Sample

With register follow-up available through 2013, we followed all children born from January 1, 1996, to November 30, 2013, with respect to a diagnosis of neonatal seizures (made in the first month of life). In the evaluation of epilepsy, we required a minimum of 2 years of follow-up, thus restricting the sample to children born before December 31, 2011.

### Measures

#### Exposure

We defined SSRI/SNRI exposure from the maternal self-report at enrollment to antenatal care, typically corresponding to the end of the first trimester. However, we used a cohort covered by the PDR (i.e., the 751,887 births from mid-2006 to 2013) to show that of the women who self-reported SSRI/SNRI use during pregnancy, 64% had filled SSRI/SNRI prescriptions in the first trimester, 35% had filled SSRI/SNRI prescriptions in the second trimester, and 34% had filled SSRI/SNRI prescriptions in the third trimester. Using the same cohort, we also found the agreement between self-reported SSRI/SNRI use and second/third trimester SSRI/SNRI filled prescriptions was 98.5% (κ 0.63, 95% CI 0.63–0.64).

SSRIs included medications with an anatomical therapeutic chemical (ATC) code beginning with N06AB (fluoxetine, citalopram, paroxetine, sertraline, fluvoxamine, escitalopram, and unspecified SSRIs). SNRIs included medications with the following ATC codes: N06AX16 (venlafaxine) and N06AX21 (duloxetine). In our cohort, 23,160 (1.49%) children were born to women who reported use of at least 1 SSRI or SNRI (Table 1).

**Table 1:**
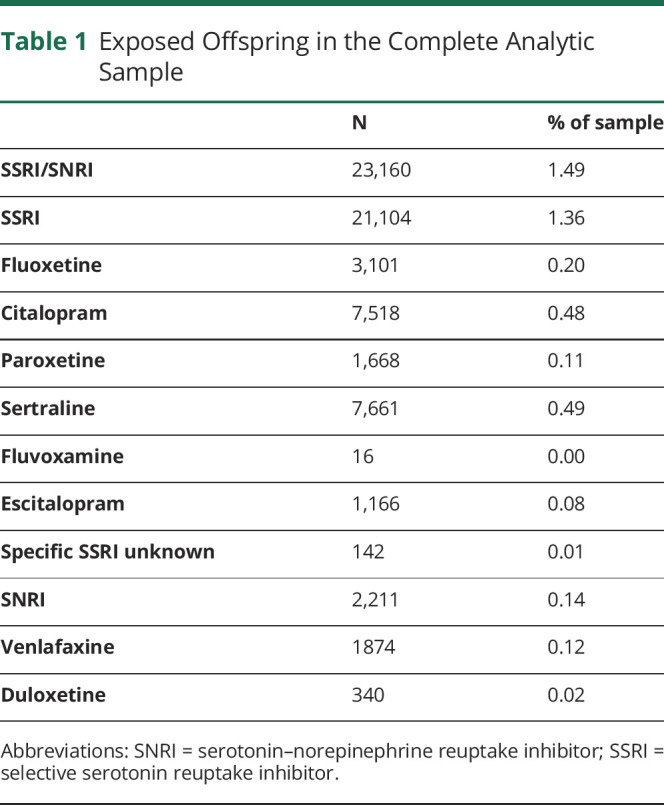
Exposed Offspring in the Complete Analytic Sample

#### Outcomes

Neonatal seizures were identified via at least 1 diagnosis (ICD-9,779.0 and ICD-10 P90) in the first 30 days of life.^1-13,19^ Diagnosis of epilepsy (ICD-9 345 and ICD-10 G40-G41) was identified using the same sources at any time after birth.

#### Covariates

Covariates were identified for their ability to block potential influence from hypothesized common causes of maternal antidepressant use during pregnancy and seizure disorders in children. Pregnancy-specific characteristics included the year of birth, maternal self-report of tobacco smoking, and other psychotropic medication use at the first antenatal visit. Individual parental characteristics included age and highest level of education at the time of birth and diagnosis of epilepsy, mood disorders, anxiety disorders, substance use disorder, schizophrenia, bipolar disorder, suicide attempt, or record of criminal convictions prior to conception. At the family level, we considered sociodemographic factors, including parental cohabitation status, income, and a measure of neighborhood deprivation in the year of the birth. The neighborhood deprivation scores incorporated annual proportions of welfare recipients, unemployed, immigrants, divorced individuals, and individuals with low educational attainment and measures of residential mobility, crime rates, and neighborhood disposable income.^31^

### Data Analytic Plan

We analyzed data for this study using the SAS software system, version 9.4. We first performed descriptive analyses on our identified covariates, exposures, and outcomes. We then estimated associations (unadjusted and adjusted for covariates) between maternal SSRI/SNRI use in pregnancy and children's risk of seizures, using log-binomial regression for the cumulative incidence of seizures in the neonatal period and Cox proportional hazard regression for incident diagnosis of epilepsy during follow-up. We followed children from birth to first diagnosis or censoring at time of emigration, death, or end of follow-up as the underlying time scale.

After estimating the overall associations, we adjusted for maternal indications for antidepressant use (i.e., mood and anxiety disorders) to examine the specific influence of confounding by indication. Next, we further adjusted for maternal and paternal history of epilepsy prior to conception to partially examine the potential influence of genetic confounding, given that research suggests epilepsy is heritable.^32^ Finally, we estimated associations with full adjustment for all covariates, including measures of other individual parental characteristics, family sociodemographic factors, and pregnancy-specific characteristics.

We conducted a sensitivity analysis to examine whether findings were susceptible to bias related to left censoring of our data, as we were not able to capture outpatient diagnoses prior to 2001. Specifically, we re-estimated associations in a cohort of children born after 2000. We also conducted a sensitivity analysis to re-estimate associations in a cohort that excluded children exposed to multiple antidepressants during pregnancy.

## Results

Starting out with all children born from 1996 through 2013 (n = 1,781,353) we sequentially excluded those who were stillborn (n = 6,335), had invalid maternal identifiers (n = 478), were from multiple gestations (n = 52,164), had missing gestational age (n = 1,101), or had missing sex (n = 1), resulting in a cohort of 1,721,274 children. By further exclusion of children with missing information on the covariates of interest (n = 169,368), the complete case sample represented 90% of the target, with 1,551,906 children available for the examination of neonatal seizures and 1,367,087 children for the examination of epilepsy.

We first examined the distribution of relevant pregnancy (Table 2), maternal (Table 3), paternal (Table 4), and family-level (Table 5) characteristics in relation to maternal self-reports of SSRI/SNRIs. This information is provided for the target sample to also show the distribution of missing data, which ranged from none to approximately 5%. Women who reported use of serotonergic antidepressants in pregnancy were more likely to also report smoking and use of other psychotropic medications. Not only were psychiatric disorders and epilepsy more common among these women, they were also more common in their partners (Tables 3 and 4).

**Table 2:**
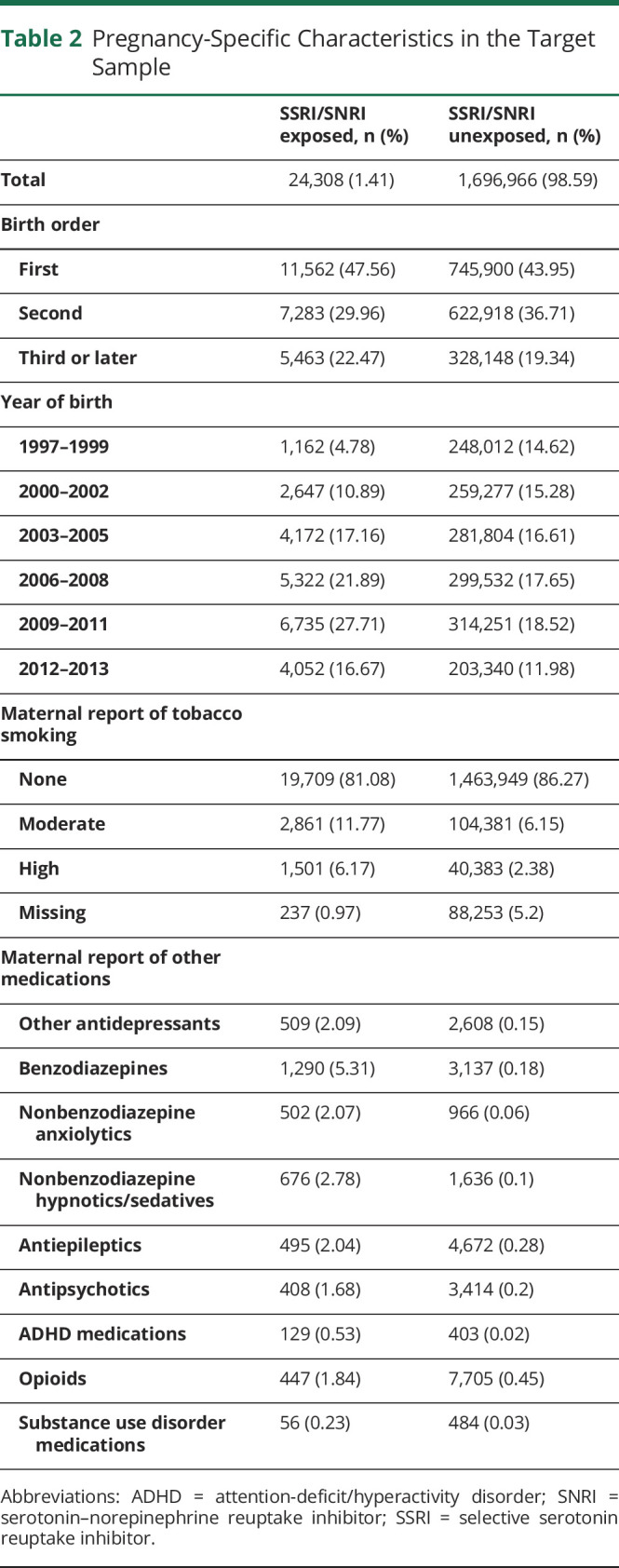
Pregnancy-Specific Characteristics in the Target Sample

**Table 3:**
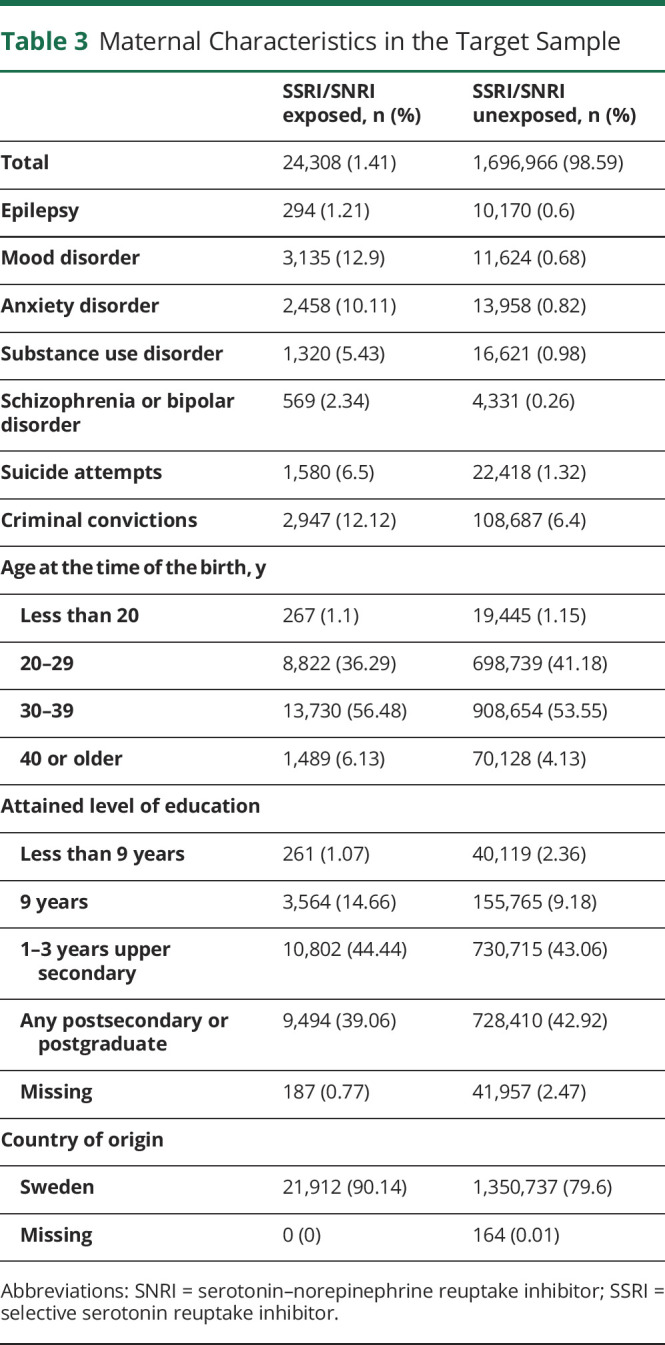
Maternal Characteristics in the Target Sample

**Table 4:**
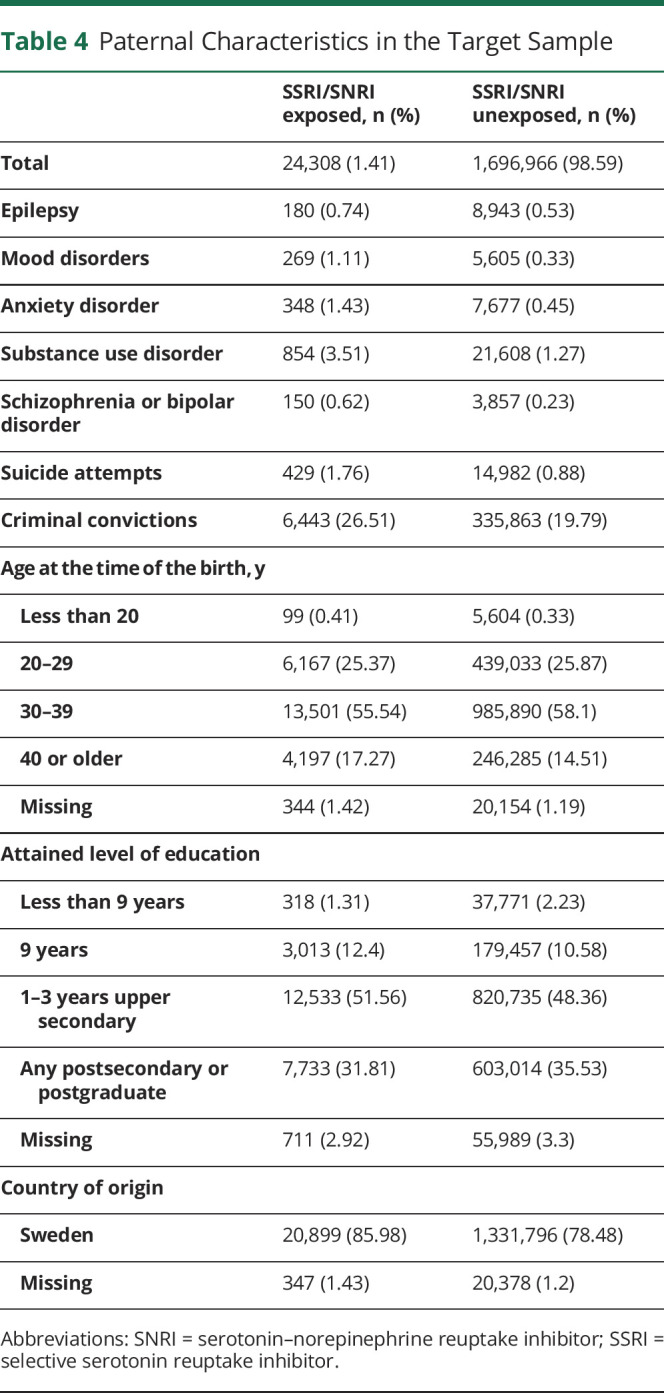
Paternal Characteristics in the Target Sample

**Table 5:**
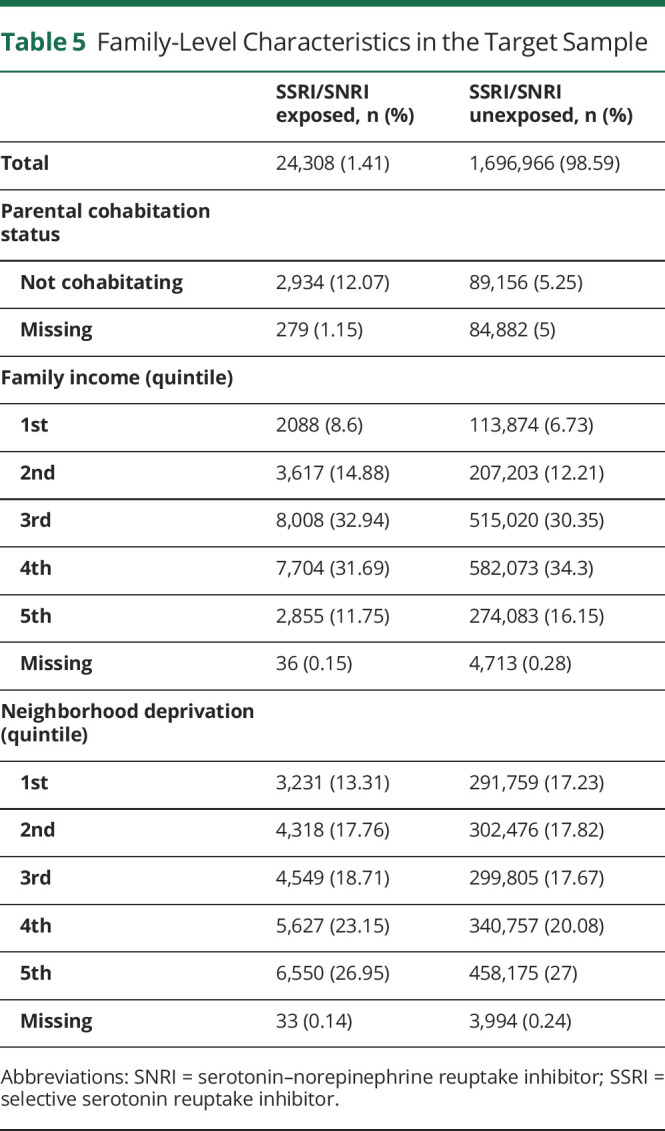
Family-Level Characteristics in the Target Sample

In the full analytic sample, 1.36% of women reported use of SSRIs. The most commonly reported medications were sertraline (0.49%), citalopram (0.48%), and fluoxetine (0.20%). Fewer women (0.14%) reported use of SNRIs in pregnancy, with venlafaxine being the most commonly reported medication (0.12%; Table 1).

Documented occurrence of seizures in the first month of life were rare, but more common among exposed than unexposed children (1.7 vs 1.2 per 1,000). By age 5 years, 5.4 per 1,000 exposed and 4.1 per 1,000 unexposed children had been diagnosed with epilepsy (Table 6).

**Table 6:**
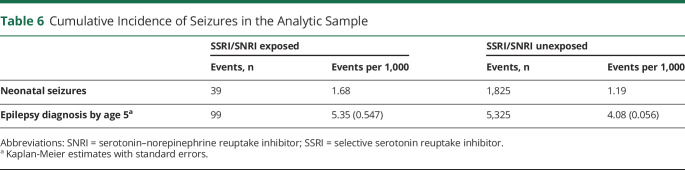
Cumulative Incidence of Seizures in the Analytic Sample

Children born to mothers who reported use of SSRIs or SNRIs in pregnancy had 41% greater risk of experiencing seizures in the newborn period (risk ratio [RR] 1.41, 95% CI 1.03–1.94) and 21% greater risk of being diagnosed with epilepsy during follow-up (hazard ratio [HR] 1.21, 95% CI 1.03–1.43) compared with children whose mothers did not report such use (Table 7). Adjustment for maternal indications for SSRI/SNRI use led to some attenuation of the observed associations (RR 1.30, 95% CI 0.94–1.80; HR 1.13, 95% CI 0.95–1.33). Further adjustment for maternal and paternal history of epilepsy had little to no influence on the estimates of association, whereas adjustment for remaining covariates greatly attenuated the remaining associations (RR 1.10, 95% CI 0.79–1.53; HR 0.96, 95% CI 0.81–1.14; Table 7).

**Table 7:**
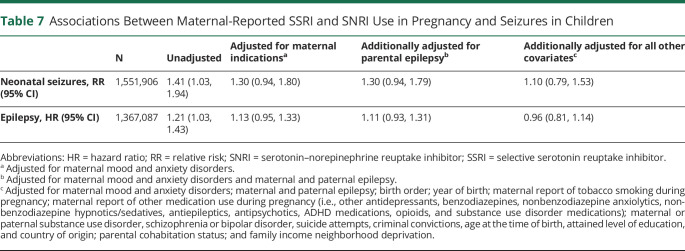
Associations Between Maternal-Reported SSRI and SNRI Use in Pregnancy and Seizures in Children

Findings from fully adjusted sensitivity analyses on a restricted subsample of children born after 2000 suggest that our main findings were not influenced by the left censoring of our data (eTable 1, links.lww.com/WNL/B971). Our main findings were not affected by the inclusion of children exposed to >1 antidepressant in pregnancy (eTable 2, links.lww.com/WNL/B972).

This study provides Class II evidence that exposure to SSRIs/SNRIs in the first trimester of pregnancy is not associated with an increased incidence of neonatal seizures and epilepsy.

## Discussion

Using a large nationwide sample, and with adjustment for a more extensive set of confounding factors than previous research,^19^ we examined the risk of seizure disorders in children whose mothers reported use of SSRIs and SNRIs in pregnancy. Our evaluation of 2 seizure outcomes—neonatal seizures and childhood epilepsy—yielded overall reassuring results.

Consistent with prior research,^1-13^ we observed a higher risk of neonatal seizures among children exposed to SSRIs/SNRIs in pregnancy, with 0.17% of exposed children affected compared with 0.12% of unexposed children. We added to prior research by showing that this association was almost entirely explained by systematic differences between the groups (i.e., maternal indications for SSRIs and SNRIs and a wide range of other pregnancy, parental, and socioeconomic factors).

We further expand on previous literature by examining the risk of epilepsy in children born to mothers reporting SSRI and SNRI use in pregnancy. Our findings are consistent with the only other study that has investigated this association,^14^ as we observed an elevated risk that was explained by maternal indication for SSRI and SNRI use, parental history of epilepsy, and our other covariates. Thus, we did not find evidence that maternal use of SSRI and SNRI in pregnancy increases children's risk of epilepsy.

These findings are consistent with prior evidence of shared risk for seizures and psychiatric/behavioral health conditions, particularly depression.^20-22,29,30^ Research has also indicated that these comorbidities are likely to be at least partially genetically driven,^30^ such that one could expect children of parents with psychiatric and behavioral health problems to have higher risk of seizures due to genetic liability. This may explain why adjustment for these factors led to attenuation of associations in our study. In fact, our findings (1) highlight the importance of adjusting for confounding by indication (i.e., depression and anxiety disorders) and other comorbidities and (2) suggest that past research may have overestimated the risk by not adequately adjusting for confounding factors.^2,4,7-13,18^

The findings of this study are of clear clinical importance. Pregnancy is a stressful time, and the addition of depression, anxiety, and other mental health conditions add to this burden. As such, these findings may provide respite and reassurance to women (and providers) considering the risks and benefits of medication treatment, especially for those who do not have the time or resources to pursue nonpharmacologic treatment. This is even more pertinent when considering the broader research literature that has observed (1) similar null findings for other adverse outcomes in children (e.g., attention-deficit/hyperactivity disorder, autism spectrum disorder) in relation to antidepressant use in pregnancy^33^ and (2) that untreated depression and anxiety in pregnancy is related to adverse outcomes in children.^34^

This study is subject to several limitations. Although much of the existing literature examining neonatal seizures related to antidepressant use has documented strongest associations with exposure towards the end of pregnancy^19^ and suggests that exposure late in pregnancy may have important implications for longer-term neurodevelopmental outcomes,^35-37^ our exposure definition was based on maternal self-reports in the first trimester. Whereas use of maternal reports could result in some misclassification of medication use, using maternal reports would also result in less misclassification than prescription records for individuals who fill prescriptions but do not use medications. We also demonstrated high agreement between maternal reports and filled prescriptions for SSRIs/SNRIs in the first as well as later trimesters of pregnancy, which is important for 2 reasons. First, the high concordance demonstrated in this and other studies using Swedish data suggest that maternal self-report of antidepressant use is reliable.^38,39^ Second, it indicates that women reporting use in the first trimester are likely to continue use throughout pregnancy. Nonetheless, future work should also explore the timing of exposure with comprehensive adjustment for confounding factors. In addition, we did not have detailed clinical information to identify whether a diagnosis of neonatal seizures was confirmed via EEG. Whereas the potential for misclassification is not expected to be differential with respect to exposure (i.e., maternal use of SSRI in pregnancy), random misclassification could bias the estimate of association toward the null, thus hampering our ability to detect a potential small association. Our data also cannot rule out the possibility that prenatal exposure to SSRIs or SNRIs might pose risk in some individuals with specific co-occurring conditions (e.g., mosaic mutations in one's sodium channels). More research is needed to explore and understand any effect modification. Finally, future research is needed to understand the use of additional medications and polypharmacy in pregnancy and its relation to seizure outcomes in children.

Although the current study found that children of women who use SSRIs/SNRIs are at elevated risk of neonatal seizures and epilepsy, this appears to be largely due to background factors rather than to the medication use itself.

## Glossary

ATC: anatomical therapeutic chemical
HR: hazard ratio
ICD: International Classification of Diseases
PDR: Prescribed Drug Register
RR: relative risk
SNRI: serotonin–norepinephrine reuptake inhibitor
SSRI: selective serotonin reuptake inhibitor
